# Supplementary material for: Is Continuous Positive Airway Pressure a Valid Alternative to Sildenafil in Treating Sexual Dysfunction among OSA Patients? A Systematic Review and Meta-Analysis
Source: Medicina (Kaunas). 2023 Jul 17;59(7):1318. doi: 10.3390/medicina59071318 (PMC10384051; doi:10.3390/medicina59071318)
Supplement: Supplementary file 1 [file medicina-59-01318-s001.zip › medicina-2464757-supplementary.pdf]

Table S1. Grade assessment tool for bias evaluation

Study	Risk of Bias	Inconsistency	Indirectness	Imprecision	Publication Bias	Overall Quality
Perimenis et al. 2004	Unclear	Moderate	Direct	Imprecise	Unclear	Low
Perimenis et al. 2007	Unclear	Moderate	Direct	Imprecise	Unclear	Low
Pastore et al. 2014	Low	Moderate	Direct	Imprecise	Unclear	Moderate
Pascual et al. 2018	Low	Moderate	Direct	Imprecise	Unclear	Moderate
Tokgoz et al. 2015	Unclear	Moderate	Direct	Imprecise	Unclear	Low
Soner et al. 2020	Unclear	Moderate	Direct	Imprecise	Unclear	Low
Khafagy et al. 2012	Unclear	Moderate	Direct	Imprecise	Unclear	Low
Taskin et al. 2010	Low	Moderate	Direct	Imprecise	Unclear	Moderate
**Summary Judgements**	Mostly unclear	Predominantly moderate	Largely direct	Generally imprecise	Largely unclear	Mixed low and moderate
